# 24 h movement behavior and metabolic syndrome study protocol: A prospective cohort study on lifestyle and risk of developing metabolic syndrome in undergraduate students from low-income regions during a pandemic

**DOI:** 10.3389/fepid.2022.1010832

**Published:** 2022-09-29

**Authors:** Marcus Vinicius Nascimento-Ferreira, Kliver Antonio Marin, Ruhena Kelber Abrão Ferreira, Luiz Fernando Oliveira, Ana Caroline Bandeira, Paula Silva Sousa, Josilene Miranda de Sousa, Antonio Gibran de Almeida Cardoso, Lorrane Cristine Conceição da Silva, Ana Clara Arrais Rosa, Millena Vaz de Carvalho, Ithamara Sthefanny Pereira de Carvalho Silva, Alaiana Marinho Franco, Francisco Leonardo Torres-Leal, Heráclito Barbosa de Carvalho, Augusto César Ferreira de Moraes

**Affiliations:** ^1^Health, Physical Activity and Behavior Research (HEALTHY-BRA) Group, Universidade Federal do Tocantins, Miracema, Brazil; ^2^Youth/Child Cardiovascular Risk and Environmental (YCARE) Research Group, Faculdade de Medicina, School of Medicine, University of São Paulo, São Paulo, Brazil; ^3^Instituto de Ensino Superior do Sul do Maranhão (IESMA/UNISULMA), Imperatriz, Brazil; ^4^Metabolic Diseases, Exercise and Nutrition Research Group (DOMEN), Department of Biophysics and Physiology, Centre for Health Sciences, Federal University of Piaui, Teresina, Brazil; ^5^Michael & Susan Dell Center for Healthy Living, Department of Epidemiology, Human Genetics and Environmental Science, The University of Texas Health Science Center, Houston School of Public Health (UTHealth School of Public Health), Austin Campus, Austin, TX, United States

**Keywords:** metabolic diseases, physical activity, sedentary behavior, sleep time, COVID-19

## Abstract

**Introduction:**

Obesity and its comorbidities are increasingly prevalent in Latin America, with a more rapid growth in individuals with lower income. The composition of movement behaviors within a 24 h period may have important implications for obesity, metabolic and mental health in cross-sectional data. However, a longitudinal study is needed to confirm the findings from the primarily cross-sectional evidence. The COVID-19 pandemic has been associated with cardiometabolic outcomes and has impeded healthy behavior.

**Objectives:**

The first objective is to evaluate the time elapsed since the diagnosis of not meeting 24 h movement guidelines and the potential subsequent onset of metabolic syndrome in undergraduate students from low-income regions within 4 years of follow up. The second objective is to test the association between 24 h movement, mental wellbeing, eating behaviors, and abdominal obesity in the period of this pandemic.

**Methods:**

The 24 h movement behavior and metabolic syndrome (24 h-MESYN) study is a multicentre cohort study that will include participants from two Brazilian cities within the 2022–2025 period to asses the first objective, and also a nested case-control study at the baseline will be carried out to evaluate the second objective. Previously, we conducted a feasibility study in the academic year of 2021 to assessing the psychometric properties of subjective tools, refine our study protocol, and adjust the epidemiological conditions of the cohort's subsequent phases (like as prevalence of exposure of interest, sampling process, and study adherence). Statistical tests as Cohen's kappa agreement; factorial analysis; logistic, Poisson and linear regression; and Kaplan-Meier analysis will be performed, in accordance with the objectives.

## Introduction

Obesity and its comorbidities are increasingly prevalent in Latin America, with a more rapid growth in individuals with lower income (1), and have a central role in metabolic syndrome (2). In this line, the prevalence of metabolic syndrome in Latin America is around 14% to 27% in adults (3); whereas, an extensive systematic review indicated that the prevalence of this outcome is between 28.9 and 29.6% in Brazilian adults depending on the diagnostic criteria adopted (4) following a worldwide trend (2). The prevalence of metabolic syndrome appears to be lower in university students ranging from 0 and 19.2% (5) reaching 20.5% in low-income Brazilian university students (6). Genetic predisposition, smoking, aging, proinflammatory state and hormonal changes have a causal effect on this outcome (4) so does lifestyle (2, 4).

The composition of movement behaviors within a 24 h period may have important implications for obesity and metabolic health (7). The entire 24 h day is composed of four main movement behaviors: light-intensity physical activity, moderate-to-vigorous-intensity physical activity, sedentary behavior and sleep time (7–9). Hence, movement behaviors can be considered to be codependent and compositional: a change of time spent in one movement behavior necessitates an exchange of equal time for one or a combination of other movement behaviors (8, 9). Findings from a recent systematic review indicate that the time-use composition of these behaviors in a 24 h period is associated with indicators of adiposity at all ages (7). In addition, the pediatric population who fulfill all of the 24 h movement guideline recommendations generally have more favorable measures of adiposity, mental, social, emotional, and cardiometabolic health than those who do not fulfill these recommendations (7).

The COVID-19 pandemic led to an unprecedented health crisis, with a great impact on 24 h movement behavior, as well as political and economic crises. This pandemic has greatly impacted Latin America, after first affecting China and other Asian countries and then Europe and North America (1). Studies have associated the current pandemic period with cardiometabolic (1) and mental (10) health, and with a deterioration of healthy behaviors (11, 12). Expectedly, the literature indicates decreasing physical activity and increasing sedentary behavior in this pandemic (11, 12). Additionally, age, sex, income, occupation, obesity, and being worried about contracting COVID-19 are associated with these changes (11). Obesity (a marker of metabolic syndrome) has consistently been associated with increased COVID-19 severity (1).

On the other hand, the COVID-19 pandemic affected education in many ways and produced an urgent change in the university courses from traditional to distance learning (13). Governmental actions have adopted restrictives measures for reducing the spread of the coronavirus in this context, such as limiting social contact, suspending face-to-face teaching, and implementing online solutions wherever practicable (14). In this way, confinement changed students' learning strategies to a more continuous habit (14). The current format of university education due to the COVID-19 pandemic has affected the academic course of undergraduate (13) and graduate (13, 15) students with a negative impact on the psychological and mental health of the students, rendering them more anxious and depressed (15). In Brazil, unequal access to digital tools, connectivity and lack of training has imposed unseen challenges for governments, schools and teachers to engage students in long distance education during the COVID-19 pandemic, which reinforce the negative impact of this online education format (16).

Our study will address several key gaps in the literature. Firstly, although the association between compositional movement behaviors and cardiometabolic outcomes in young adults is well documented (7) there is no evidence regarding the association between compositional data and mental (wellbeing) health in adults (7). Secondly, there is no clear evidence that adults who fulfill the 24 h movement guidelines have better health outcomes (7). Thirdly, the literature indicates that physical activity levels decreased during the COVID-19 lockdown and sedentary behavior levels increased (11), in other words, 24 h movement guidelines meeting was low before the pandemic and even worse afterwards. Mainly this is regarding the population of Brazil (12). Fourthly, there is no evidence of the depth of the change in 24 h behavior movement longitudinally caused by the pandemic, as well as its resumption after the end of a lockdown. And, finally, this pandemic is showing how fragile the Brazilian public educational policies have historically been, where abrupt change affects all actors in the educational systems, but students with a low socioeconomic status may be hit critically (16).

Thus, we designed 24 h movement behavior and metabolic syndrome (24h-MESYN) study aiming (i) to evaluate the time elapsed since the diagnosis of not fulfilling 24 h movement guidelines and the potential subsequent onset of metabolic syndrome in undergraduate students from low-income regions within 4 years of follow up; and (ii) to test the association between 24 h movement, mental wellbeing, eating behaviors, and abdominal obesity in the pandemic.

## Materials and methods

### Study design

**Figure 1:**
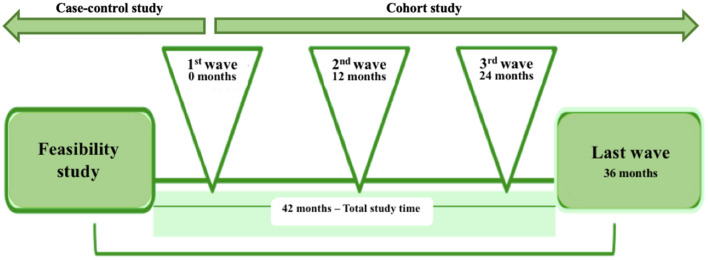
Timeline of the 24 h movement behavior and metabolic syndrome (24h-MESYN) study.

The 24h-MESYN study will be a 48-month longitudinal multicentre study (cohort follow-up), with five measurements over this period (Figure 1). The study is subdivided into three phases: a feasibility study (phase 1), a prospective cohort (phase 2) and a nested case-control study (phase 3). The feasibility study was conducted in the first half of 2021. The first wave (baseline) of the cohort should start in second half of 2022 or as soon as the Brazilian health authorities allow the return to face-to-face classes in that academic year.

### Population

The 24h-MESYN study will be conducted in two research centers, a private university of Imperatriz, Maranhão, Brazil and a public university of the city of Miracema do Tocantins, Tocantins, Brazil. The majority of their almost 3,200 undergraduate students currently come from the surrounding small cities in Maranhão (e.g., Açailandia, Estreito and Porto Franco) and the state of Tocantins (e.g., Lajeado, Miranorte, and Tocantínia) and regions across the north and northeast (e.g., the state of Para), with a lesser number of students coming from other regions in the country. Both cities are in the poorest states of the country, with a Gini index of 0.56 and 0.43, for Imperatriz and Miracema do Tocantins, respectively (17). At the start of the academic year of 2020, the private university informed us that 2,225 students were registered, enrolled in 9 undergraduate programs (Business Administration, Law, Physical Education, Nursing, Aesthetics and Cosmetics, Physiotherapy, Nutrition, Psychology, and Social Work); whereas the public university informed us that there were 968 students enrolled in four undergraduate programs (Physical Education, Pedagogy, Psychology, and Social Work).

### Ethics

The 24h-MESYN study was approved by the Research Ethics Committee (CEP, ID: 4,055,604 and 5,161,340). The project follows international ethical principles for research with humans (i) Declaration of Helsinki, revised in 2008, Seoul, Korea; and national ethical principles (ii) resolution of CNS 466/12 and (iii) guidelines for conducting research activity during the pandemic caused by the COVID-19 (available at: http://www.fo.usp.br/wp-content/uploads/2020/07/Orientações-condução-de-pesquisa-e-atividades-CEP.pdf) and (iv) orientations for research in a virtual environment (OFÍCIO CIRCULAR N° 2/2021/CONEP/SECNS/MS).

After the institution's written consent, the selected students will receive a formal and detailed invitation letter about risks, benefits, objectives and methods of the study, where they will be able to voluntarily consent to collaborate with the project. Participants who agree to participate in the study and sign (virtually in phase 1 and physically in the subsequent phases) the informed consent form will undergo informative clarifications about the days and data collection.

### Methodology harmonization and fieldwork training

A premise of this multicentre study is to ensure that the data collection is performed in a standardized way, thereby achieving a good representation of reality and allowing comparisons of the data from the different cities involved in the study (18). In our study, the data collection will be conducted by two different fieldwork teams based on harmonized methodology. These teams will conduct subjective and objective measurements in their respective study centers, where each team will have a local coordinator who will be responsible for monitoring all data collection. The final number of researchers in these teams depends on the possibilities of each center, with a minimum of four researchers from health sciences, among undergraduate and graduate students (at least one). The data management system will be centralized and coordinated by the general coordinator of the study at the Federal University of Tocantins. In addition, standardized procedures will be adopted to optimize the accuracy and consistency of the findings obtained during the study, such as manuals and standardized measurements, records protocols, and training and supervision of researchers.

The 24h-MESYN study has two field work training programs planned. The training programs were designed by an experienced scientist who has participated in two other multicentre studies in Europe (19) and South America (18, 20). Prior to the feasilibity study (phase 1), the researchers were given 20 h of training in field work to obtain the necessary qualifications to carry out the data collection with the subjective instruments (e.g. giving questionnaires, online messages standardization, data control and tabulation). In this program, the researchers also reviewed all subjective instruments, searching for orthographic and formatting errors. In addition, prior to the cohort study (phase 2), the researchers will undergo further fieldwork training. The research team will undergo a training program to collect data *via* objective instruments. They will perform triplicate measurements on 10 participants (*per* field worker). These data collection sessions will include: anthropometric measures and blood pressure (18). This training will not involve blood collection.

### Study protocol

#### Feasibility study (phase 1): Sample, data collection, and eligibility criteria

As phase 1, we conducted a feasibility study in order to identify the proportion of students who fulfill the 24 h movement behavior guidelines (21); as well as the psychometric properties of the subjective instruments, their reliability (temporal stability) and the construct validity. Here, all students answered the questionnaire twice, the second time after a two-week interval. For the sample size estimation, we adopted the assumptions of Nascimento-Ferreira et al. (22). The parameters used to calculate the sample size were: α = 0.05, β = 0.20 (power of 80%) and a correlation coefficient of 0.28 (22). Based on these parameters, we estimated a sample of 98 students. Anticipating losses of 50.0%, rejections of 50.0% and missing data of 25.0% (18) in each test-retest assessement, we decided to invite 342 ([2.5 × 98] + 98 ≅ 343) students to participate. At the design level, participants were distributed in a proportion of 60/40 by sex (female and male) and program of study (health and other sciences) based on previous cohorts (6, 23).

However, we carried out our sampling protocol only in the Maranhão research center due to national and regional health protocols and recommendations related to COVID-19. Students, in this phase, were randomly selected and invited to participate presencially. We delivered a link with the questionnaire to students who agreed to participate, and up to three reminders were sent in the subsequent initial invitation and t two-week intervals. Students provided informed consent to participate anonymously by completing and submitting the questionnaire in *Google Forms* (available at: https://forms.gle/L92wXsVaxxfPNgpE8). All data were self-reported electronically.

We all participants included in the study were enrolled in the university, 17 years or older, and had signed the informed consent form. Students who reported incomplete data were excluded from the analysis of the corresponding instrument (e.g. incomplete data from vigorous physical activity leads to the exclusion of that answer to the questionnaire from the reliability and validity analysis).

#### Cohort study (phase 2): Sample, data collection, and eligibility criteria

In phase 2, we will conduct a longitudinal study to evaluate the time of fulfilling 24 h movement behavior guidelines (or not) and its relation with metabolic syndrome (21). For the prospective cohort, the parameters used for sample size estimation were α = 0.01, β = 0.10 (or 90% power), prevalence of fulfilling 24 h movement behavior guidelines ranging from 7.7% (during the pandemic, Table 1) to 23.1% (in a non pandemic period (9)), precision 5% and design effect of 2.0 (multistage sampling) for a two-sample proportions test. The estimated sample size was 200 in each research center, we corrected this estimation for population ratio (2,225/968 = 2.3) achieving a minimum sample size of 279 and 121 students from the private (Maranhão research center) and the public (Tocantins research center) institution. We add 125.0%, anticipating potential losses, rejections and missing data. Thus, we will invite 502 students (349 from the private and 152 from the public institution).

**Table 1:** Overview of measurements and variables.

**Method**	**Measurement of interest**	**Variables**
Anthropometric	Abdominal obesity and nutritional status	Waist circumference, weight and height
Omron automatic monitor	Blood pressure and heart rate	Systolic, diastolic blood pressure and heart rate
Blood samples	Biological markers in fasting venous	Triglycerides, high density lipoprotein (HDL) cholesterol and fasting blood glucose
Questionnaire	Physical Activity	Light, moderate and vigorous physical activities
	Sedentary Activities	Screen time and sedentary behavior
	Sleep	Sleep quality, habits and time
	Mental wellbeing	Perceived Stress and satisfaction with life
	Eating behaviors	Food restriction, emotional intake and external intake
	Sociodemographic and economics characteristics	Age, biological sex, ethnicity, marital status, neighborhood, socioeconomic status, maternal education, personal and family income and daily workload
	Academic environment	Program study area, shift, time, number of classes enrolled and time spent studying for classes
	COVID-19 burden	Perceived knowledge, sources of information, behaviors, and academic and life difficulties

In each institution, the sample will be selected by random sampling in two stages, program and students. Thus, a list of all students from up to second semester offered will requested. From this list in each stratum a simple random sampling will be carried out, with probability proportional to the number of students in each institution study program and shift (e.g., morning and afternoon) in relation to the total number of students. Then, in each selected program of studies and shift, students will be randomly invited to participate in the study. The probability of selection for each student will be condionted on the number of students in these two stratums and an additional invitation rate of 30.0% for males.

We will collect the data in four visits *per* wave. In the first visit, we will conduct an explanation of the 24h-MESYN study objectives/methodology and we will delivery the informed consent form. As this study phase will occur presencially, the student will sign two copies of the informed consent form (one for the student and another one for the researcher). In the second visit, after returning the signed form, participants will answer the questionnaires eletronically and will undergo an anthropometric and blood pressure assessment. In the third visit, a blood sample will be collected. In the fourth visit, we will deliver the results to the students.

We will adopt the same eligibility criteria as for phase 1. However, other inclusion criteria will be added: students must be regularly enrolled up to second semester and present in the classroom on the day of the objective measurements. We will exclude participants who fail to provide complete information about age, biological sex, program of studies, shift and time (period). In addition, only those students who answer the questionnaire and participate in the anthropometric measurements will be qualified for the blood sample collection. Students who report a physical disability will be evaluated, but they will be excluded from the analysis.

#### Case-control study (phase 3): Sample, data collection, and eligibility criteria

In phase 3, we will conduct a case–control study in the cohort first wave to test associations between 24 h movement, mental wellbeing, eating behaviors (exposures), and abdominal obesity (main outcome) and in a subgroup of students with the data collected in the baseline (21). Students with prevalent abdominal obesity will be selected as a case. We will adopt four controls *per* case and they will be paired by sex and age (±1 year) (24).

### Variables

The variables that will be measured are described in Table 1.

#### Main outcome

Metabolic syndrome will be based on the International Diabetes Federation criteria (2), which establish the presence of any 3 of 5 risk factors: presence of abnormal waist circumference (≥ 90 cm in men; ≥ 80 cm in women), blood pressure (systolic ≥ 130 and/or diastolic ≥ 85 mmHg), triglycerides (TG, ≥ 150 mg/dL), HDL cholesterol (HDL-c, ≤ 40 mg/dL in men; ≤ 50 mg/dL in women) and fasting blood glucose (≥ 100 mg/dL).

We will evaluate the waist circumference according to international standards (25) with an inelastic tape, at the midpoint between the lower edge of the last rib and the top iliac crest (26). A third measurement will only be performed in case of error greater than 5% between the first and second measurement. The assessments will be carried out in a private room at the institution. All measurements will be taken in underwear or with as little clothing as possible and no shoes (18).

Blood pressure measurements will be performed following the recommendations of the American Heart Association (27). We will perform measurements with the Omron HEM-7320-LA Automatic Arm Oscillometric Device (Omron Healthcare, Kyoto, Japan), with a pressure range of 0–299 mmHg and a heart rate range of 40–180 beats/minute. Blood pressure and heart rate will be measured twice, with an interval of 2 min between the two measurements, according to international guidelines. If the values of the second measurement vary by more than 5% from the first, a third measurement will be taken (28).

A blood venous sample will be collected using a Vacutainer system (Becton Dickinson, Oxford, UK). This procedure will be carried out in the morning, after an 8–12 h overnight fast. Participants will be instructed to fast on the morning of the collection day. Samples will be collected and analyzed by a previously accredited laboratory following international protocols and recommendations (18), such as: do not perform moderate-to-vigorous physical activities and do not consume products with caffeine and alcoholic beverages and avoid drinking water in the hours preceding the assessments.

#### Exposures

The exposures will be 24 h movement behavior, mental wellbeing (perceived stress and satisfaction with life) and eating behaviors. These exposures will be subjectively measured by self-adminstered validated questionnaires. All subjective instruments are available at *Google Forms* (https://forms.gle/L92wXsVaxxfPNgpE8).

We will adopt two techniques for assessing 24 h movement behavior data, compositional data and fulfillment of the guidelines (7). The compositional data will be assessed by the total time spent in physical activity (light and moderate-to-vigorous), sedentary behavior and sleep time during a 24 h period (7). Additionally, we will classify 24 h movement behavior guidelines into fulfilling or not fulfilling the following criteria: (1) ≥150 min per week of moderate-to-vigorous of physical activity; (2) ≤ 8 h per day of sedentary time (No more than 3 h per day of recreational screen time); (3) sleeping 7–9 h per day; and (4) adhering to all three guidelines (29).

Physical activity will be measured *via* the International Physical Activity Questionnaire, short version (30). A instrument that assesses physical activity by questions about frequency, duration and intensity of activities (light, moderate and vigorous), including activities performed during leisure and as a form of transport. Sedentary behavior will be assessed *via* the South American Youth Cardiovascular and Environmental (SAYCARE) sedentary behavior questionnaire (31). An instrument composed by 10 questions about time spent on weekdays and weekends, in activities such as watching television; playing games on a computer, cell phone or tablet; computer use; studying, reading books or magazines; and driving a car, bus or train (31). In addition, we will apply the Longitudinal Aging Study Amsterdam Sedentary Behavior Questionnaire (LASA-SBQ) addressing average time spent watching television, using a computer, sitting, talking, napping, among other sedentary behaivours, during weekdays, and weekend days through 20 items (32). Sleep quality, habits and time will be measured *via* the Pittsburgh Sleep Quality Index (33). This is a self-administered 19-question questionnaire that assesses seven components of sleep (sleep quality, sleep latency, sleep duration, habitual sleep savings, sleep changes, sleep changes, use of medications for sleep, and sleep dysfunction) (33).

Perceived stress will be assessed by the Perceived Stress Scale (34), a scale based on 14 questions with answer options ranging from 0 to 4 (0 = “never” to 4 = “always”). Questions with a positive connotation (4–7, 9, 10, 13) have their summed score inverted. The other questions are negative and must be added directly. The scale total is the sum of the scores for all these 14 questions. Satisfaction with life will be assessed using the Satisfaction with Life Scale (35), a questionnaire that assesses the cognitive component of life satisfaction, through five items, with response options ranging from 1 (strongly disagree) to 7 (strongly agree), given that the closer to 7 the response, higher are the life satisfaction ratings.

Eating behaviors (psychosocial aspects) will be measured *via* the Dutch Eating Behavior Questionnaire (DEBQ) (36). The questionnaire consists of 33 items, evaluated on a scale rated between 1 and 5 (never/rarely/sometimes/often/veryoften), comprising three subscales: food restriction (10 items); emotional intake (13 items); and external intake (10 items).

#### Other covariables

We will retrieve variables about sociodemographic and economic characteristics, academic environment and COVID-19 (perceived) burden using self-admininstered instruments. We will also assess the body mass index objectively.

Sociodemographic variables will be based on questions addressing biological sex, age, ethnicity, marital status and neighborhood (37). Socioeconomic status will be assessed by the Brazilian Economic Classification Criteria (38), a self-administered instrument that uses a point system for possession of goods and education of the family head, classified into A to E, with A being the most educated and wealthy and E the least. The questionnaire estimates the purchasing power of individuals and families. In addition, we will include questions addressing academic environment: maternal education (incomplete high school, high school, technical education, university degree), personal and family income (in minimum wages) and daily workload (hours *per* day).

Regarding the academic environment, the following data will be collected: undergraduate study program area, shift, time (semester), number of classes enrolled (in the semester) and time spent studying for classes (hours *per* day). For the COVID-19 burden, we will retrieve data from knowledge, sources of information, behaviors, and academic and life difficulties *via* a questionnaire based on the fact sheets of the Centers for Disease Control and Prevention (CDC) (39). A total of 10 questions, for seven of which the answer can be true or false, while three questions will assess the level of agreement with the statements on a 5-point scale (0 = “strongly disagree” to 4 = “agree totally”). And, for the body mass index, we will measure weight (kg) and height (cm). The body mass index will be calculated using the standard formula (BMI = weight/height^2^).

### Statistical analysis

We will perform all statistical analyses using Stata version 15.0 (Stata Corporation, College Station, TX, USA). We will consider *p* values ≤ 0.05 statistically significant. The normality of the variables will be evaluated using the Shapiro-Wilk test.

#### Descriptive analysis

Continuous variables will be described by their means and standard deviations. Categorical variables will be described by absolute and relative frequency, and 95% confidence interval (40). In addition, we will use time series to describe the trend of the variables. A descriptive analysis will identify the time elapsed between exposure to 24 h movement behavior to a metabolic syndrome event. The Kaplan-Meier curve will be computed.

#### Psychometric properties

To test the reliability, we will use the intraclass correlation coefficient (parametric) or Spearman correlation coefficients (non-parametric) and kappa agreement coefficient (for temporal stability), and Cronbach's alpha (internal consistency) (40). To test the validity, we will use an exploratory factor analysis (structural validity) with varimax rotation. Previously, we will use Bartlett's KMO and sphericity test to verify the adequacy of the factor analysis (40).

#### Inferential analysis

**Figure 2:**
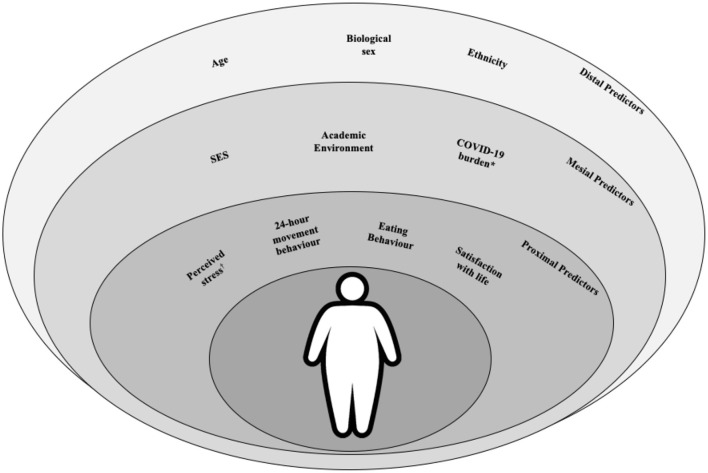
Metabolic syndrome conceptual predictors for university students in a pandemic period. SES, Socioeconomic status. *Knowledge, sources of information, behaviors, and academic and life difficulties. Also adopted as outcome for assessing the secondary objective.

Our conceptual model is presented in Figure 2. For outcome variables that are continuous, we will apply multilevel linear regression models to estimate the beta coefficient of the exposures of interest (40). For categorized variables in the case-control analysis (phase 3), we will apply a logistic regression. In addition, for categorical outcome variables, Poisson multilevel regression models with robust variance to estimate the prevalence ratios (cross-sectional data) and the relative risk (longitudinal data) will also applied (40). In both multilevel regressions, the contextual variable will be the research center. For choosing the type of multilevel model, we will use the Durbin-Wu-Hausmann method to determine random or fixed intercept model. We will analyse the fit using the Hosmer-Lemeshow test. In bivariate analysis, we will adopt a significance value of 80% (*p* ≤ 0.20).

## Preliminary results of feasibility study (phase 1)

A total of 342 students were invited to participate in phase 1 of the 24h-MESYN study. However, we gave the same questionnaire in two rounds, Q1 and Q2, with samples of 195 and 117 students, respectively. We did not identify any differential bias between Q1 and Q2 in the sensitivity analysis (*p* > 0.05). We observed a response rate of 57.0% for Q1 and 60.0% for Q2. We also observed an association between study participation and being female (PR: 0.70 [CI 95%: 0.50–0.98]; Table 2). Regarding 24 h behavior movement, we found that the proportion of students who fulfilled the guidelines was 7.7% (*n* = 15).

**Table 2:** Descriptive characteristics, sensitivity analysis and prevalence ratio of study adherence.

**Variables**	**Invited (*n* = 342)**	**Participants, Q1 (*n* = 195)**	**Participants, Q2 (*n* = 117)**	Sensitivity analysis*	**Prevalence Ratio of study adherence^†^ (95% CI)**
	**%**	**%**	**%**		
**Biological sex**				0.36	
Male	31.3	25.4	27.4		Ref.
Female	68.7	74.6	72.6		0.70 (0.50–0.98)
**Age**				0.63	
≤ 20 y		23.8	26.7		
21–25 y		44.0	45.7		
26–30 y		18.7	14.7		
31–35 y		7.3	5.2		
≥ 36 y		6.2	7.8		
**Program (study area)**				0.71	
Health Sciences	66.0	65.8	64.4		Ref.
Other areas	34.0	34.2	35.6		1.0 (0.71–1.41)
**Program shift**				0.92	
Morning		19.8	20.5		
Evening		0.5	0.9		
Night		62.0	62.4		
Integral		17.7	16.2		
**Program time**				0.73	
≤ 3rd semester	27.9	24.9	29.3		Ref.
> 3rd semester	72.1	75.1	70.7		0.82 (0.58–1.16)
**Meeting 24 h movement guidelines** ^**a**^					
No		92.3	92.3	0.99	
Yes		7.7	7.7		

CI, confidence interval; Q1, questionnaire first application; Q2, questionnaire second application. Significance differences (p < 0.05) are in bold. ^*^p-value, goodness-of-fit test for comparison between the sample in the first and second questionnaire application. ^†^Outcome: not acceptance (43.6%). ^a^Adhering to all three guidelines.

## Discussion

The 24h-MESYN study was designed by experienced researchers to investigate the composition of movement behaviors, within the course of a 24 h period during the pandemic, of undergraduate students from low-income regions and their associations with other behaviors and health outcomes based on longitudinal data. Preliminary achievements in phase 1 of the study were to improve our data collection skills, refine the study procedure, data retrieval and tabulation, and adjusting the epidemiological conditions to design the cohort's subsequent phases (e.g., prevalence of exposure of interest, sampling process, study adherence).

Our sample size inflation, around 125.0% (for each measurement), was sufficient to obtain the designed sample size. This is not in line with the literature, which suggests an additional sample of 10.0–15.0% for potential refusals and/or losses (41). One potential explanation could be that those from a low-income region have decreased partipation rates in studies with questionnaires. A recent multicentre study carried out in a South American pediatric population showed that socioeconomic status was a limiting factor for study adherence among adolescents and mothers (who answered the children's questionnaires) (18). We believe that this can be extrapolated to our results.

On the other hand, an American (11) and a European (23) cohort study conducted with undergraduate students identified the majority of the students to be female. In addition, the European cohort also identified the majority of its students as coming from the health sciences program area (23). This is in line with our findings from the feasibility study. However, our findings indicate that there is a better adherence to study participation from females, which indicates an additional invitation rate for male of around one-third in this population. And, our feasibility study also indicates a low proportion of students who fulfill the 24-h movement behavior guidelines, which is not far from previous findings in the pandemic, as a prior cohort study with Brazilian adolescents found proportion of around 7.5% (12). A comprehensive cohort study found an adults' observed compliance rate of 23.1% in a non-pandemic period (9).

We have not received any complaints regarding the questionnaire's format or length. However, we speculate that our decreased partipation rate in Q2 can be attributable to the length of the questionnaire. We could possibly explain this due to a decrease in the participants' motivation to complete a second questionnaire within a short lead time.

## Ethics statement

The studies involving human participants were reviewed and approved by the Research Ethics Committee of Universidade Ceuma (ID: 4.055.604) and Universidade Federal do Tocantins (ID: 5.161.340), respectively. The students/participants provided their written informed consent to participate in this study.

## Author contributions

MN-F and KM were responsible for study coordination, article design, statistical analysis, and scientific writting. RA, LO, AB, PS, JM, AC, LS, AA, MC, IP, and AF were resposible for data collection, article design, and scientific writing. MN-F, FT-L, HB, and AF were responsable for study design, article design, statistical analysis, and scientific review. All authors contributed to the article and approved the submitted version.

## Funding

The 24h-MESYN study design, data collection and analysis were supported by the Brazilian Government from National Counsel of Technological and Scientific Development (CNPq; proc. 402391/2021-7). MN-F received a postdoctoral scholarship from Programa Nacional de Pós-Doutorado/Capes (PNPD/CAPES). LO and LS received a scientific initiation scholarship from the National Council for Scientific and Technological Development (CNPq; proc. 72041/2022-1 and CNPq; proc. 116316/2022-5). AA received a scientific initiation scholarship from the Institute of Higher Education of Southern Maranhão (IESMA/UNISULMA). AF was supported by UTHealth School of Public Health Start-up Funding.

## Conflict of interest

The authors declare that the research was conducted in the absence of any commercial or financial relationships that could be construed as a potential conflict of interest.

## Publisher's note

All claims expressed in this article are solely those of the authors and do not necessarily represent those of their affiliated organizations, or those of the publisher, the editors and the reviewers. Any product that may be evaluated in this article, or claim that may be made by its manufacturer, is not guaranteed or endorsed by the publisher.
